# Biocompatibility Assessment of a Hybrid Material Based on Epoxy-Treated Pericardium and Polyvinyl Alcohol Cryogel in a Rat Vascular and Subcutaneous Implantation Model

**DOI:** 10.17691/stm2026.18.3.03

**Published:** 2026-06-30

**Authors:** E.A. Ovcharenko, T.V. Glushkova, E.A. Senokosova, K.Yu. Klyshnikov, T.N. Akentieva, A.A. Klyueva, A.V. Mironov, A.E. Kostyunin

**Affiliations:** 1 PhD, Head of Laboratory of Novel Biomaterials; Research Institute for Complex Issues of Cardiovascular Diseases, 6 Academician Barbarash Blvd., Kemerovo, 650002, Russia; 2 PhD, Senior Researcher, Laboratory of Novel Biomaterials; Research Institute for Complex Issues of Cardiovascular Diseases, 6 Academician Barbarash Blvd., Kemerovo, 650002, Russia; 3 PhD, Head of Laboratory of Cellular Technologies; Research Institute for Complex Issues of Cardiovascular Diseases, 6 Academician Barbarash Blvd., Kemerovo, 650002, Russia; 4 PhD, Researcher, Laboratory of Novel Biomaterials; Research Institute for Complex Issues of Cardiovascular Diseases, 6 Academician Barbarash Blvd., Kemerovo, 650002, Russia; 5 Junior Researcher, Laboratory of Novel Biomaterials; Research Institute for Complex Issues of Cardiovascular Diseases, 6 Academician Barbarash Blvd., Kemerovo, 650002, Russia; 6 Junior Researcher, Laboratory of Genomic Medicine; Research Institute for Complex Issues of Cardiovascular Diseases, 6 Academician Barbarash Blvd., Kemerovo, 650002, Russia; 7 PhD, Junior Researcher, Laboratory of Cellular Technologies; Research Institute for Complex Issues of Cardiovascular Diseases, 6 Academician Barbarash Blvd., Kemerovo, 650002, Russia; 8 PhD, Senior Researcher, Laboratory of Novel Biomaterials; Research Institute for Complex Issues of Cardiovascular Diseases, 6 Academician Barbarash Blvd., Kemerovo, 650002, Russia

**Keywords:** bioprosthetic heart valves, structural valve degeneration, epoxy-treated bovine pericardium, polyvinyl alcohol, biocompatibility, rat model, hybrid material

## Abstract

**The aim of the study** was to evaluate the biocompatibility of a hybrid material based on epoxy-treated bovine pericardium and cryostructured polyvinyl alcohol (PVA) after implantation into the rat aortic wall and subcutaneous tissue.

**Materials and Methods:**

The study evaluated hybrid material patches based on epoxy-treated bovine pericardium and cryostructured PVA; unmodified “KemPeriplasNeo” pericardial patches served as controls. The samples were implanted into the abdominal aortic wall and subcutaneously in male Wistar rats. The vascular implantation periods were 5 and 20 days, and the subcutaneous implantation periods were 60 and 120 days. The specimens were excised from the aorta and studied histologically using Russell–Movat staining and immunostaining for neutrophil myeloperoxidase (MPO) and the macrophage marker CD68. The calcium content in subcutaneously implanted samples was assessed by spectrophotometry and alizarin red S staining. The quantitative data were presented as the median, percentiles, minimal and maximal values (Me [25%–75%; min–max]).

**Results:**

After implantation into the abdominal aortic wall, the hybrid material samples showed a lower tendency toward thrombotic deposit formation on the surface compared to the pericardium. On day 5 of implantation, the thrombus thickness was 49.1 [34.7–64.6; 27.4–71.6] μm in the experimental group vs 170.3 [158.1–210.3; 124.4–217.0] μm in the controls (p<0.001). By day 20 of implantation, the macrophage density was lower in the peri-implant area of the modified samples compared to the controls: 219 [187–275; 112–362] vs 301 [244–338; 194–433] CD68^+^ cells per field of view (p<0.001), respectively. The neutrophil density at all experimental time points, as well as the macrophage density on day 5 of implantation did not differ significantly between the tested materials (p>0.17). No signs of calcification were detected in either group following the subcutaneous implantation over a period of 60 or 120 days. The control pericardium in the subcutaneous model showed the signs of cell-mediated degradation, whereas the hybrid samples were resistant to immune-cell infiltration and preserved their internal architecture.

**Conclusion:**

The modification of epoxy-treated bovine pericardium with cryostructured PVA cryogel improved its biocompatibility and enhanced resistance to biodegradation in a rat model after aortic-wall and subcutaneous implantation. The developed material may be used to create next-generation heart valve bioprostheses with potentially improved resistance to structural valve degeneration.

## Introduction

Nearly 400,000 cardiac valve substitutes are implanted annually, and about 80% of them are xenogeneic bioprostheses [1]. The number of valve prosthetic surgeries is steadily on the rise, and according to expert prognoses, by the middle of the current century it may reach 850,000 procedures per year [1, 2]. It is significant that the service life of bioprostheses is limited: within 15 years after implantation, up to 30% of them need to be replaced [3, 4]. The primary cause of bioprosthetic valve dysfunction is structural valve degeneration (SVD) — a gradual and irreversible process characterized by the accumulation of degenerative changes (biological tissue rupture and calcification) in the valvular apparatus [5]. Thus, due to a limited lifetime, the increasing use of bioprostheses in global cardiac surgery will inevitably result in more prosthetic dysfunctions. Since no effective techniques have been developed to slow down or prevent SVD, the problem of limited bioprosthetic durability remains urgent [6, 7].

One promising direction in the search for approaches to protect bioprostheses against SVD is treating biological tissue with polymer solutions (e.g., polyethylene glycol or polyvinyl alcohol — PVA) to form biocompatible gels [8, 9]. When biological tissue interacts with a polymer, it results in the formation of a hybrid material consisting of a biological component and a synthetic component. Collagen fibers act as a reinforcing matrix of a hybrid material, while the gel coating them prevents circulating molecules in the recipient’s blood from penetrating into the resulting material, and also prevents the invasion by cellular elements of the immune system [8–11].

Previously, the authors described the original modification of the epoxy-treated bovine pericardium based on its treatment with liquid PVA solution followed by cryostructuring [9]. The technology enhanced the biomaterial resistance to calcification and proteolysis in an *in vitro* experiment without impairing its mechanical properties and hemocompatibility [9, 11]. The hybrid material samples also exhibited high resistance to leukocyte adhesion under simulated conditions of pulsatile blood flow in Ibidi Pump System Quad (Ibidi GmbH, Germany) using special chambers [10].

The present study continued the safety and performance assessment of the developed hybrid material.

**The aim of the study** was to evaluate the biocompatibility of a hybrid material based on epoxy-treated bovine pericardium and cryostructured PVA after implantation into the rat aortic wall and subcutaneous tissue.

## Materials and Methods

### Biomaterial under study

The study material consisted of fragments of bovine pericardial patches “KemPeriplasNeo” (KPi7080M; CJSC “NeoCor”, Russia) modified with cryostructured PVA according to the original technique. We used 15% PVA solution (341584; Sigma-Aldrich, USA) obtained by dissolving PVA in deionized water at 95–100°С under constant stirring. The bovine pericardial patches were immersed for 24 h in the prepared solution cooled to room temperature, then removed from the solution and placed between two glass plates, with the gap fixed by metal spacers of appropriate thickness. Subsequently, the samples underwent 3 cryostructuring cycles consisting of successive steps: the samples were first kept at −40°С for 24 h, and then at −2°С and +8°С for 12 h. The resulting modified patches were washed for 24 h in deionized water at room temperature with two water changes to remove unbound PVA.

The control group consisted of unmodified pericardial patches “KemPeriplasNeo” (KPi7080M; CJSC “NeoCor”, Russia).

### Laboratory animals and implantation techniques

All experiments were carried out on male Wistar rats. The tested material was implanted subcutaneously in the rats weighing 50–60 g, and into the abdominal aorta in the rats weighing 350–400 g. The surgeries were performed under inhalation anesthesia with a mixture of isoflurane and oxygen using EZ-7000 Classic System (EZ Systems, USA) under sterile conditions. Prior to the operation, the fur on each animal back or abdomen was shaved depending on the surgical procedure; and the implantation area was treated with skin antiseptic.

For subcutaneous implantation, two 0.5-cm incisions were made on the right back and along the left side of the spine. Subcutaneous pockets were formed from the incisions, and the samples (0.6×0.6 cm) of the tested materials were placed into the pockets. Each animal was implanted with two samples — the experimental sample and the control one (in each group of materials, one sample was taken for histology, another for calcification quantification). The incisions were sutured using non-absorbing polyester suture Lavsan 3/0 (Lintex, Russia). The implantation periods were 60 and 120 days (six animals per time point).

When implanted into the aorta, the incision was made along the abdominal midline to provide access to the abdominal aorta. Then the longitudinal dissection of the aortic wall was made, a 2×5-mm patch of the tested material (experimental or control) was sutured in place (one sample per rat) so that the patch formed the anterior vascular wall. After the surgery, the abdominal wall and the skin of the animals were sutured in layers using non-absorbing polyester suture Lavsan 4/0 (Lintex, Russia). The implantation periods were 5 and 20 days (five animals per material at each time point).

No medical treatment was given to the rats involved in the experiments described above. Upon completion of the experiments, the animals were euthanized with carbon dioxide, and the material patches were dissected together with the adjacent tissues for subsequent analysis.

### Histological and immunohistochemical staining

The samples of the tested materials with the adjacent tissues removed from rats after both subcutaneous and aortic implantation were washed in 0.9% NaCl solution, and embedded in quick-freezing tissue medium Neg-50 (6502; Thermo Fisher Scientific, USA) and then placed in liquid nitrogen. Then the samples were cut at −25°С using an HM525 cryostat microtome (Thermo Fisher Scientific, USA). The prepared 6-μm-thick histological sections were placed on glass microscope slides.

The structural changes of the tested materials and their integration with the surrounding tissues were assessed using the kits for pentachrome Russell–Movat staining (ab245884; Abcam, Great Britain) and alizarin red staining (ab142980; Abcam, Great Britain). Histological staining was performed according to the manufacturer’s protocols. The stained sections were mounted with a coverslip using mounting medium “Vitrogel” (HM-VI-A250; BioVitrum, Russia).

The intensity of the inflammatory response to sample implantation was assessed by immunohistochemical staining of the prepared sections using the antibodies against neutrophil myeloperoxidase MPO (ab208670; Abcam, Great Britain) and the macrophage marker CD68 (ab125212; Abcam, Great Britain). Prior to staining, the sections were fixed for 10 min at room temperature in 4% paraformaldehyde followed by washing (3 times for 5 min) in phosphate-buffered saline (pH 7.4) on a shaker. The immunohistochemical staining was performed using NovoLink Polymer DS (RE7150-CE; Leica Biosystems, USA) kit according to the manufacturer’s protocol. The antibodies to MPO and CD68 were dissolved in 1% bovine serum albumin solution in saline at a 1:1000 dilution. The sections were incubated with the antibodies in a closed box at 4°С for 18 h. The stained sections were mounted with a coverslip using mounting medium “Vitrogel”. The sections stained without the primary antibodies against MPO and CD68 served as controls.

The stained histological sections were scanned using an automated laboratory biological microscope MT5300L (Meiji Techno, Japan). QuPath v.0.6.0 software was used to process the histological slides and prepare images.

### Biocompatibility assessment of explanted samples

QuPath v. 0.6.0 software was used to measure the thickness of the thrombotic deposits and neointima formed on the surface of the samples implanted into the abdominal aortic wall after 5 and 20 days, respectively, and to analyze the leukocyte infiltration density in the peri-implant area [12]. For each material type, the thickness of the thrombotic deposit or neointima was assessed on 10 random histological sections (the mean value was calculated from 10 measurements for each section). The cells stained with antibodies against MPO or CD68 were counted on 5 random sections for each type of material at each time point (1 section from each experimental animal). The leukocytes in each section were counted per 5 fields of view, 200×200 μm in size, adjacent to the implanted sample.

### Calcium content determination in the samples

Calcium content in the tested materials after subcutaneous implantation was measured spectrophotometrically. The samples were separated from the adjacent tissues and washed in 0.9% NaCl solution. They were then lyophilized in a FreeZone 2.5 Plus (Labconco, USA) for 24 h, and weighed. The samples were then digested in 65% perchloric acid (0.5 ml) at 160–180°С on a sand bath LH-402 (Loip, Russia) until completely dissolved. The final volume was adjusted to 5 ml with sterile water for injection. The calcium content in the solution was determined using a Multiskan Sky spectrophotometer (Thermo Fisher Scientific, USA) at a wavelength of 575 nm using a commercial Calcium Assay Kit (ab102505; Abcam, Great Britain) according to the manufacturer’s instructions.

### Statistical analysis

The statistical analysis was performed using GraphPad Prism 8 (GraphPad Software, USA). Due to the small sample size, we used nonparametric statistical methods. Mann–Whitney U-test was applied for the between-group comparisons. The differences were considered significant if p<0.05. The quantitative data were presented as the median, percentiles, minimal and maximal values (Me [25%–75%; min–max]).

### Ethics statement

The experiments involving laboratory animals were carried out in accordance with the principles of the European Convention for the Protection of Vertebrate Animals used for Experimental and Other Scientific Purposes (Strasbourg, 1986). Animal experiments were approved by the local ethics committee of the Research Institute for Complex Issues of Cardiovascular Diseases (Kemerovo, Russia) (protocol No.11, approval date: October 13, 2025).

## Results

The study of the samples of the tested materials (the hybrid material based on epoxy-treated bovine pericardium and cryostructured PVA, and the control epoxy-treated bovine pericardium) implanted into the rat abdominal aortic wall showed a similar response of the surrounding tissues. Specifically, 5 days after implantation, loose fibrin deposits composed of accumulations of nucleated cells were detected on the luminal surface of samples from both groups (Figure 1). After 20 days, a thin neointimal layer consisting of loosely packed collagen fibers with inclusions of fibrin and mucopolysaccharides, as well as the connective tissue cells with spindle-shaped nuclei, was observed over fragments of the tested materials (see Figure 1).

**Figure 1.**
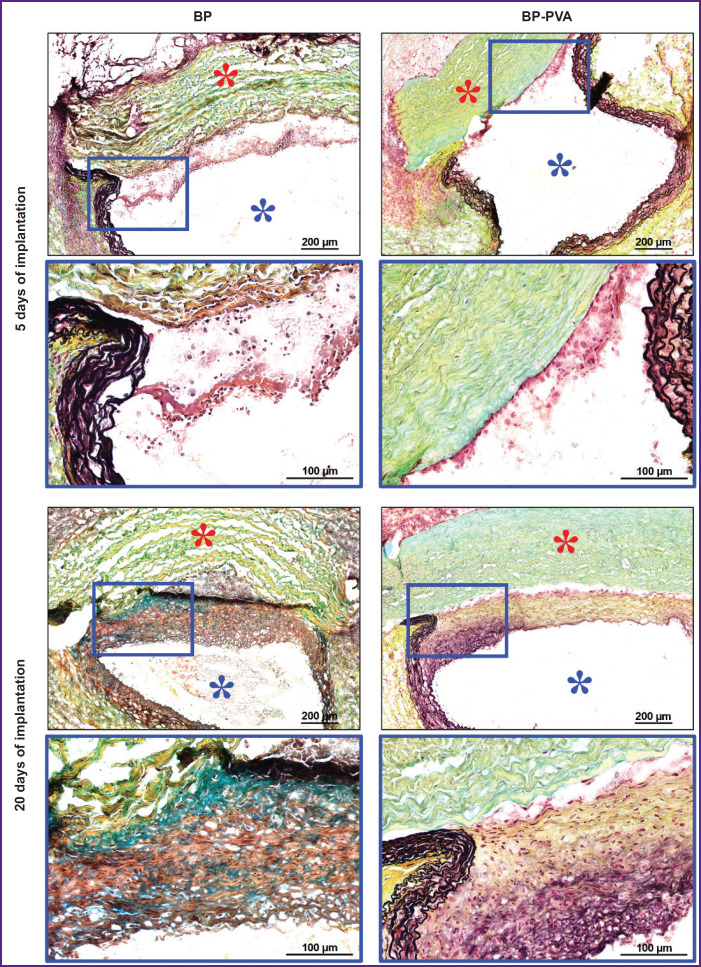
Histological images of the control epoxy-treated bovine pericardial (BP) samples and the hybrid material based on epoxy-treated bovine pericardium and cryostructured polyvinyl alcohol (BP-PVA) implanted into the rat abdominal aortic wall (pentachrome staining according to Russell–Movat) On day 5 and day 20 of implantation, thrombotic deposits and neointima, respectively, were observed on the surface of both materials. Red staining indicates fibrin, yellow — collagen, black — elastic fibers, dark green — mucopolysaccharides, burgundy — cell nuclei. In the presented images the red asterisk indicates the sample, a blue asterisk — the vascular lumen. Magnification 100× and 400× for overview images and high-magnification images, respectively

The semiquantitative analysis showed a tendency toward less pronounced formation of thrombotic deposits on PVA-modified bovine pericardium than on the control pericardium (Figure 2 (a)). The thickness of the thrombi in the first case was 49.1 [34.7–64.6; 27.4–71.6] μm, in the second case — 170.3 [158.1–210.3; 124.4–217.0] μm (p<0.001). However, neointimal thickness did not differ between the study groups reaching 168.1 [133.1–181.6; 34.8–196.4] μm and 176.8 [150.4–244.1; 128.1–337.0] μm (p=0.58) when formed on the surface of the hybrid material and the pericardium, respectively (Figure 2 (b)).

**Figure 2.**
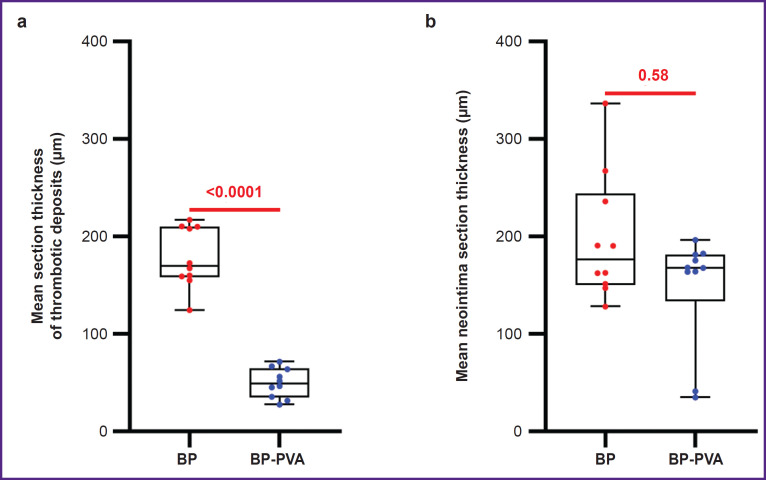
Semiquantitative thickness of thrombotic deposits (a) and neointima (b) formed on the surface of the tested materials implanted into the rat abdominal aortic wall BP — control epoxy-treated bovine pericardium; BP-PVA — hybrid material based on epoxy-treated bovine pericardium and cryostructured polyvinyl alcohol

It should be noted that at both time points, day 5 and day 20, the tested materials were found to preserve their initial structure showing few signs of collagen fiber breakdown. However, after 20 days, cellular infiltrates were found deep within the control pericardium, while the samples of the hybrid material had none.

Immunohistochemical staining showed the accumulation of neutrophils (MPO^+^) and macrophages (CD68^+^) in the peri-implant area of the studied samples. The thrombotic deposit formed on the surface of the tested samples, after 5 days of implantation, was rich in neutrophils, although there were no neutrophils in the neointima on day 20 of the experiment (Figure 3). The largest accumulations of neutrophils were detected close to the sample margin, along the suture line, at both 5 and 20 days after implantation.

**Figure 3.**
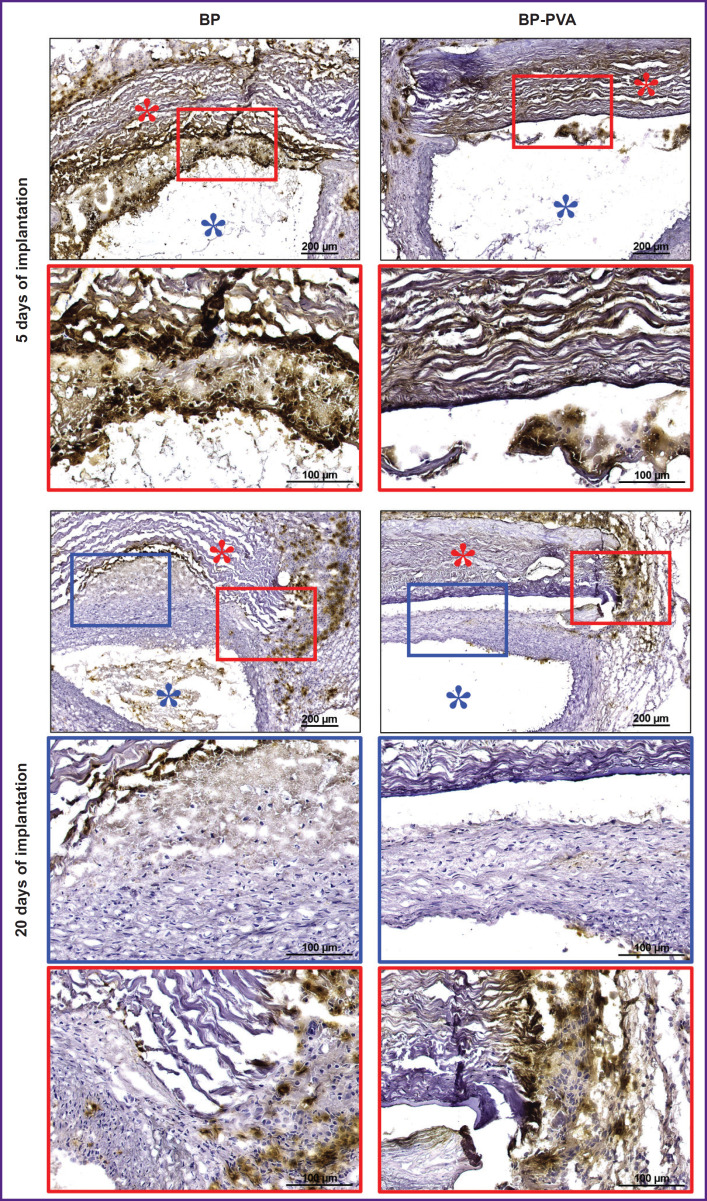
Examples of control epoxy-treated bovine pericardial (BP) samples and the samples of the hybrid material based on epoxy-treated bovine pericardium and cryostructured polyvinyl alcohol (BP-PVA) implanted into the rat abdominal aortic wall (immunohistochemical staining using antibodies against neutrophil myeloperoxidase) The sample is marked by a red asterisk, a blue asterisk indicates the vascular lumen, a positive signal is shown by brown staining. Magnification 100× and 400× for overview images and high-magnification images, respectively

The macrophage localization on day 5 of implantation was characterized by marked heterogeneity in both groups of the materials under study: there were single cells and small accumulations of cells distributed relatively evenly throughout the peri-implant area, and there were single cells as part of thrombotic deposits (Figure 4). On day 20 of the experiment, the macrophages were found to be concentrated primarily at the boundary between the implanted sample and the surrounding tissues; and the macrophages were scarce in the neointima (see Figure 4). Moreover, macrophage penetration was observed deep into the control pericardium accompanied by the initial fragmentation and stratification of collagen fibers in the samples. No signs of macrophage invasion were found inside the hybrid material fragments.

**Figure 4.**
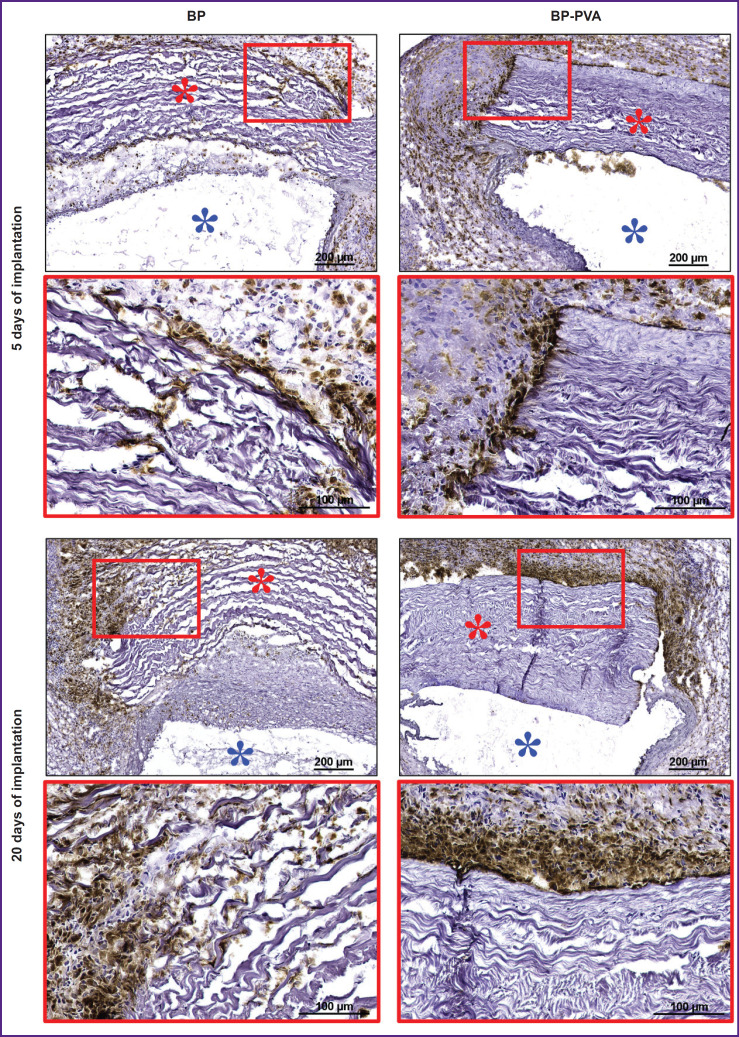
Images of the control epoxy-treated bovine pericardial (BP) samples and the samples of the hybrid material based on epoxy-treated bovine pericardium and cryostructured polyvinyl alcohol (BP-PVA) implanted into the rat abdominal aortic wall (immunohistochemical staining using antibodies against the macrophage marker CD68) The sample is marked by a red asterisk, a blue asterisk indicates the vascular lumen, a positive signal is shown by brown staining. Magnification 100× and 400× for overview images and high-magnification images, respectively

The semiquantitative assessment of cellular infiltration was performed to better characterize an inflammatory response developing when the rats underwent implantation with fragments of epoxy-treated pericardium and its modified variant in the aortic wall. The analysis findings showed that the number of neutrophils in the peri-implant area of the control and the experimental samples did not differ after 5 days (52 [32–77; 12–101] vs 49 [20–70; 7–92] MPO^+^ cells per field of view, respectively; p=0.50), or after 20 days (45 [15–105; 2–243] vs 77 [33–133; 14–202] MPO^+^ cells per field of view, respectively; p=0.17) (Figure 5 (a, b)). Similarly, the number of macrophages on day 5 of the experiment was similar in both groups (73 [44–90; 25–149] CD68^+^ cells per field of view for the pericardial fragments and 68 [55–89; 29–161] — for the hybrid material samples; p=0.94), however, on day 20 after implantation the control samples demonstrated more pronounced infiltration than the experimental ones (301 [244–338; 194–433] vs 219 [187–275; 112–362] CD68^+^ cells per field of view, respectively; p<0.001) (Figure 5 (c, d)).

**Figure 5.**
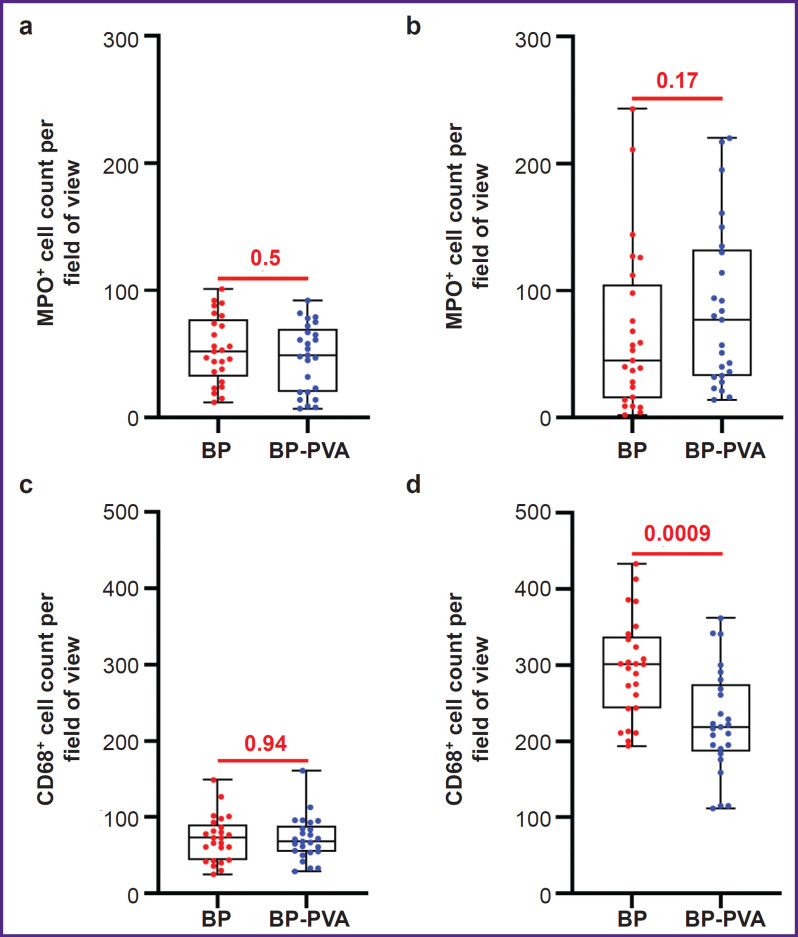
Semi-quantitative assessment of cell infiltration in the peri-implant area of the tested materials after implantation into the rat abdominal aortic wall: (a, b) neutrophil count after 5 and 20 days of implantation, respectively; (c, d) macrophage count after 5 and 20 days of implantation, respectively. BP — control epoxy-treated bovine pericardium; BP-PVA — hybrid material based on epoxy-treated bovine pericardium and cryostructured polyvinyl alcohol

To assess the susceptibility of the tested materials to calcification, their fragments were subcutaneously implanted in rats. Regardless of the implantation period (60 or 120 days), no calcifications were detected histologically in either group (Figure 6). The quantitative assessment also showed low calcium content in both groups of the materials under study and in both implantation periods, with no significant differences between them: 2.81 [2.25–3.47; 1.79–3.55] vs 3.65 [2.91–5.99; 2.67–6.25] mg calcium/g of dry tissue after 60 days of implantation (p=0.13), and also 3.13 [2.82–4.68; 2.26–5.13] vs 4.11 [2.47–5.41; 2.25–6.32] mg calcium/g of dry tissue after 120 days of implantation (p=0.82) for the control pericardium and the hybrid material, respectively (Figure 7).

**Figure 6.**
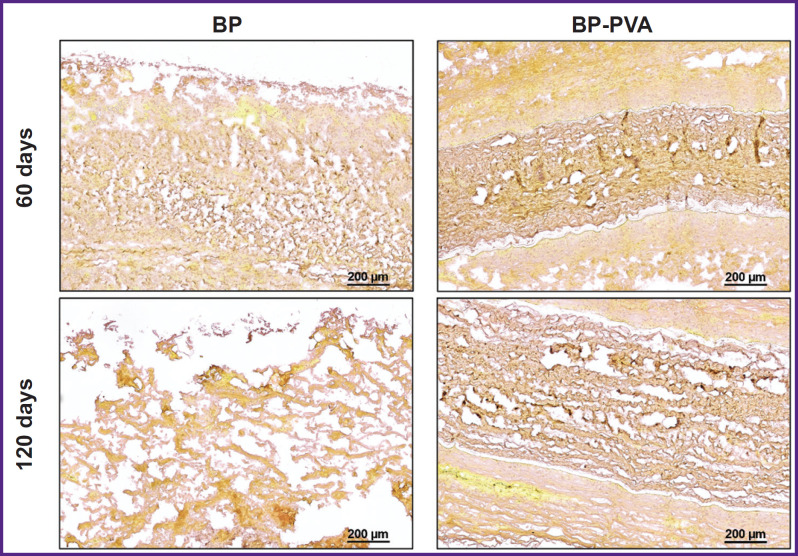
Images of the control epoxy-treated bovine pericardial (BP) samples and the samples of the hybrid material based on epoxy-treated bovine pericardium and cryostructured polyvinyl alcohol (BP-PVA) subcutaneously implanted in rats (alizarin red S staining) No calcifications are detected at either implantation period. Magnification 100×

**Figure 7.**
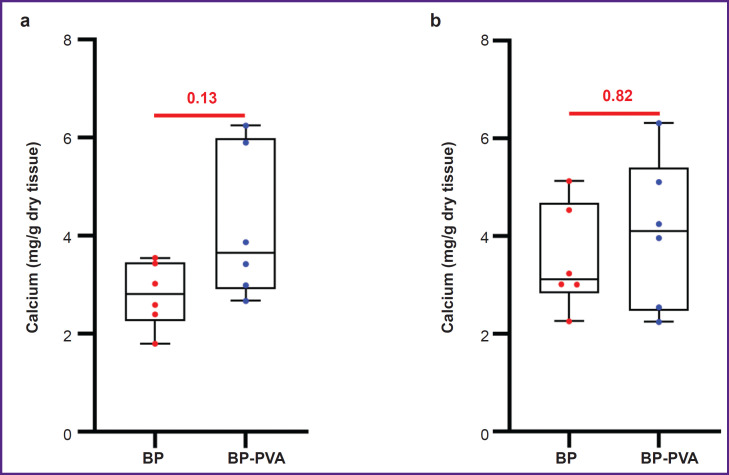
Quantitative assessment of calcium content in the tested materials subcutaneously implanted in rats after 60 (a) and 120 days (b) BP — control epoxy-treated bovine pericardium; BP-PVA — hybrid material based on epoxy-treated bovine pericardium and cryostructured polyvinyl alcohol

It should be noted that the histological analysis showed that samples of the control material removed after subcutaneous implantation exhibited the signs of fragmentation and loosening of collagen fibers (Figure 8). Moreover, the boundary between the epoxy-treated pericardium and the connective tissue capsule surrounding the sample was poorly distinguishable. Macroscopically, the control fragments appeared thickened, and the described condition could be observed on both 60 and 120 days after implantation. The samples stained with antibodies against CD68 showed that the observed collagen damage resulted from extensive macrophage invasion of the extracellular matrix of the samples. However, the hybrid material fragments appeared to preserve an intact internal structure, and were clearly distinguishable from the surrounding tissues. The macrophage infiltrates were localized at the implant periphery, not penetrating deep into the material (see Figure 8).

**Figure 8.**
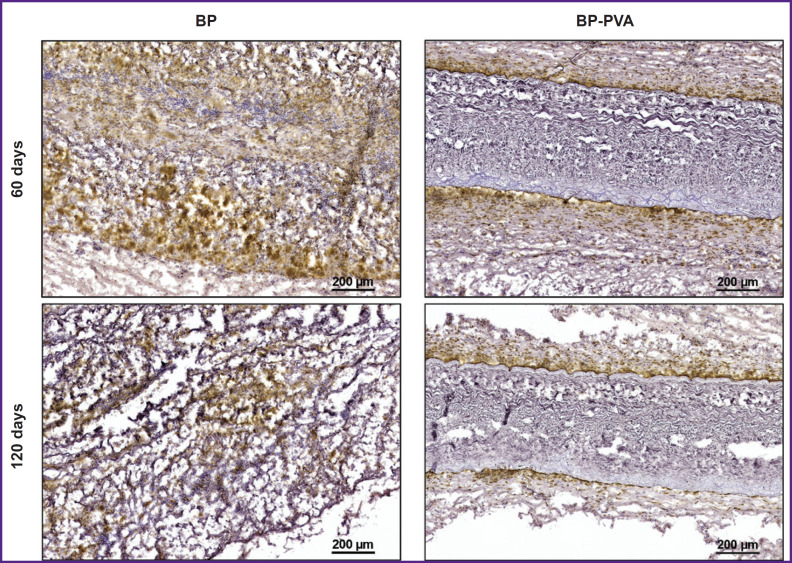
Images of the control epoxy-treated bovine pericardial (BP) samples and the samples of the hybrid material based on epoxy-treated bovine pericardium and cryostructured polyvinyl alcohol (BP-PVA) subcutaneously implanted in rats (immunohistochemical staining using the antibodies against the macrophage marker CD68) An intense positive signal is present (brown staining) deep in the control samples, and there is no macrophage invasion in the hybrid material. Magnification 100×

## Discussion

Calcification and cell-mediated inflammation in bioprosthetic leaflets are considered the primary causes of SVD; however, no approach currently provides complete protection against these processes [13–15]. The method we propose is based on treating the bovine pericardium with PVA solution followed by cryotreatment; the method made it possible to obtain the hybrid material, which demonstrated promising results in *in vitro* experiments [9–11]. Specifically, the hybrid material accumulated three times less calcium during 6-week incubation in a calcifying solution compared to the commercial pericardium, and it also resisted collagen fiber damage during 24-hour treatment with bacterial collagenase [9, 11]. Additionally, the protective PVA layer on the modified biomaterial surface reduced the adhesion of platelets and leukocytes under *in vitro* blood-flow modeling conditions [9, 10].

The present study findings are in good agreement with the previous findings [9–11]. Thus, the hybrid material implanted into the rat abdominal aortic wall showed a lower tendency toward thrombotic deposit formation on the surface than the bovine pericardium in the control group. This is most likely due to the anti-adhesive properties of PVA cryogel as a component of the hybrid material [10].

This aspect is relevant to the protection against SVD. Although the recipients of bioprostheses rarely develop symptomatic thrombosis of an implant (it develops postoperatively in 1–2% of operated patients), the subclinical thrombus formation on the surface of the valve apparatus occurs more frequently (in nearly 10–15% of valves) [16]. Moreover, asymptomatic thrombosis can develop even several years after bioprosthetic implantation, which may have pathogenetic importance for SVD development [14]. Specifically, thrombotic deposits can contribute to reduced leaflet mobility and increased mechanical stress in the biomaterial [17]. Moreover, fibrin deposits facilitate the adhesion of leukocytes and bacterial agents, circulating in the bloodstream, to bioprosthetic valves in transient bacteremia [14, 18]. Further, it can result in the inflammation activation in bioprosthetic tissues, and in certain cases it can lead to the valve infection and the development of prosthetic endocarditis [14, 18]. Considering the above, it is reasonable to suppose that the manufacture of bioprostheses from the developed hybrid material can reduce the risk of thrombotic and infectious complications in cardiac valve replacement.

In a rat aortic implantation model, we demonstrated that the proposed pericardium modified with cryostructured PVA improved the biocompatibility of the material. This is evidenced by the lower density of macrophages in the peri-implant area of hybrid samples compared to the control fragments 20 days after implantation. Additionally, the absence of the differences in the thickness and structure of the neointima formed on the sample surface indirectly indicates comparable cytocompatibility of both material types. The reduced inflammatory response to the hybrid material is likely due to the ability of the PVA cryogel to shield immunoreactive bovine antigens in collagen fibers [14].

Interestingly, calcium accumulation in the samples of the experimental and control materials after subcutaneous implantation in rats was low, with no significant differences detected. According to literature data, epoxy-treated bovine pericardium is initially only minimally susceptible to calcification when tested in a rat model [19]. Apparently, for this reason, we did not demonstrate the advantages of the hybrid material: both materials (experimental and control) appeared to be resistant to mineralization. However, we previously showed this advantage in an *in vitro* calcification model [9]. Generally, there is reason to believe that the bovine pericardium modified with cryostructured PVA at least does not increase the biomaterial tendency toward *in vivo* calcification.

It should be noted that in both implantation models (in the vascular wall and subcutaneously) we observed no signs of damage in the hybrid material samples, while the control pericardial samples underwent infiltration by immune cells accompanied by the fragmentation of collagen fibers. Leukocytes are known to produce a wide range of matrix-degrading factors (oxygen radicals and proteases) promoting gradual degradation of both biological and polymer materials [14]. PVA cryogel serves as a barrier to immune cells and the substances they produce, thereby preserving the initial structure of a biological component of the hybrid. Given that PVA provides the resistance to cell-mediated degradation even if the samples are within rat connective tissue (where immune cells can freely come into contact with the tested material), the polymer coating may be sufficient to protect bioprostheses against leukocyte invasion under blood-flow conditions.

## Conclusion

The hybrid material based on epoxy-treated bovine pericardium and cryostructured PVA demonstrated a more favorable biocompatibility profile in a rat vascular-wall implantation model compared to the unmodified epoxy-treated bovine pericardium successfully used in clinical practice. Specifically, the direct blood contact with the experimental material resulted in the less pronounced formation of thrombotic deposits on its surface, and in the peri-implant area of the tested samples there was lower macrophage infiltration.

Taken together, these findings suggest that the developed approach based on hybridization of bovine pericardium with PVA cryogel is a potential strategy for reducing the risk of SVD. The polymer component of the biomaterial can potentially protect collagen fibers from degradation mediated by host molecular and cellular factors. Further comprehensive preclinical studies using orthotopic implantation of bioprostheses in large-animal models are required to confirm the findings.
